# Kinetic Analysis of Lidocaine Elimination by Pig Liver Cells Cultured in 3D Multi-Compartment Hollow Fiber Membrane Network Perfusion Bioreactors

**DOI:** 10.3390/bioengineering8080104

**Published:** 2021-07-23

**Authors:** Gerardo Catapano, Juliane K. Unger, Elisabetta M. Zanetti, Gionata Fragomeni, Jörg C. Gerlach

**Affiliations:** 1 Department of Mechanical, Energy and Management Engineering, University of Calabria, Via P. Bucci, I, 87030 Rende, CS, Italy; gerardo.catapano@unical.it; 2 Department of Experimental Medicine, Charité—Universitätsmedizin Berlin, Corporate Member of Freie Universität Berlin and Humboldt Universität zu Berlin, 10117 Berlin, Germany; juliane.unger@charite.de; 3 Department of Engineering, University of Perugia, 06125 Perugia, Italy; 4 Department of Medical and Surgical Sciences, Magna Graecia University, 88100 Catanzaro, Italy; fragomeni@unicz.it; 5 Department of Surgery, School of Medicine, University of Pittsburgh, & McGowan Institute for Regenerative Medicine, Pittsburgh, PA 15213, USA; joerg.gerlach@cellnet.org or; 6 Department of Bioengineering, School of Medicine, University of Pittsburgh, & McGowan Institute for Regenerative Medicine, Pittsburgh, PA 15213, USA

**Keywords:** adsorption, bioreactor, elimination, kinetics, lidocaine, liver cells, tissue engineering

## Abstract

Liver cells cultured in 3D bioreactors is an interesting option for temporary extracorporeal liver support in the treatment of acute liver failure and for animal models for preclinical drug screening. Bioreactor capacity to eliminate drugs is generally used for assessing cell metabolic competence in different bioreactors or to scale-up bioreactor design and performance for clinical or preclinical applications. However, drug adsorption and physical transport often disguise the intrinsic drug biotransformation kinetics and cell metabolic state. In this study, we characterized the intrinsic kinetics of lidocaine elimination and adsorption by porcine liver cells cultured in 3D four-compartment hollow fiber membrane network perfusion bioreactors. Models of lidocaine transport and biotransformation were used to extract intrinsic kinetic information from response to lidocaine bolus of bioreactor versus adhesion cultures. Different from 2D adhesion cultures, cells in the bioreactors are organized in liver-like aggregates. Adsorption on bioreactor constituents significantly affected lidocaine elimination and was effectively accounted for in kinetic analysis. Lidocaine elimination and cellular monoethylglicinexylidide biotransformation featured first-order kinetics with near-to-in vivo cell-specific capacity that was retained for times suitable for clinical assist and drug screening. Different from 2D cultures, cells in the 3D bioreactors challenged with lidocaine were exposed to close-to-physiological lidocaine and monoethylglicinexylidide concentration profiles. Kinetic analysis suggests bioreactor technology feasibility for preclinical drug screening and patient assist and that drug adsorption should be accounted for to assess cell state in different cultures and when laboratory bioreactor design and performance is scaled-up to clinical use or toxicological drug screening.

## 1. Introduction

The liver plays a central role in maintaining the homeostasis of human metabolism also in the presence of external challenges. To this aim, the liver performs more than 5000 important metabolic and regulatory functions, including the synthesis of plasma and coagulation proteins, the generation and accumulation of energy for the organism, the production of bile to facilitate digestion, and the metabolism of cellular waste products, drugs and xenobiotics [1]. Acute and chronic injuries to liver tissue caused by alcohol and drug abuse, poor diet, poisoning, or pathological conditions may pose a deadly threat to a patient’s life. In cases in which the pathophysiology of the injury is unknown or there is little time for pharmacologic intervention, patients need intensive extracorporeal life support and eventually orthotopic liver transplantation. In 2018, figures from the World Transplant Registry in collaboration with the World Health Organization (WHO) recorded 32,348 liver transplants performed worldwide, 7940 of which were performed in the EU. The WHO estimates that this barely covers 10% of the transplants needed in the world, pinpointing the dramatic shortage of donor organs and the need for alternative treatments to orthotopic liver transplantation [2]. Awareness is also increasing about the limits of conventional approaches to the development of new drugs. In fact, the use of animal models in the preclinical assessment of hepatotoxicity of drug candidates in many cases provides unreliable information for species-specific liver response and has serious ethical and economic implications [3]. This has prompted the quest for more reliable, sustainable and ethical in vitro cellular models as alternatives to preclinical animal models.

Engineering liver tissue in vitro by culturing liver cells in 3D perfusion bioreactors is an interesting alternative to orthotopic liver transplantation in the treatment of acute liver failure (ALF) and to animal models for preclinical in vitro pharmacological and toxicological studies. In fact, isolated liver cells possess both membranes with functioning drug transporters and phase I and phase II metabolic activities present in the liver. Biochemical cues may also be used in vitro to induce in isolated cell-specific enzymatic activities similar to the natural liver [4,5]. This makes isolated cells resemble the liver drug metabolic clearance more closely than other systems used (e.g., microsomes or isolated enzymatic preparations). Dynamic liver cell culture in three-dimensional (3D) perfusion bioreactors permits overcoming the loss of cell polarity and the de-differentiation caused by cell isolation and static 2D dish culture by promoting cell re-organization as in the natural liver. Dynamic medium supply promotes more physiological gradients of oxygen, nutrients and biochemical cues through the cell mass and waste products removal. Culture around multi-compartment networks of different membranes enables distributed integral oxygenation and CO_2_ removal. As a result, cells cultured in the most promising 3D perfusion bioreactor designs are viable and exhibit liver-specific functions for up to a month to an extent that depends on the type of cells used (e.g., single cell culture vs. co-culture), the scaffold geometry, architecture, and physical-chemical properties, and the bioreactor design and operation (e.g., decentralized oxygen supply, medium perfusion through cell aggregates, etc.) [6,7].

Cell culture in the 3D multi-compartment perfusion bioreactors with integral membrane oxygenation proposed for bioartificial livers (BALs) or to engineer liver tissue in vitro is expected to foster the expression of liver-specific enzymatic activities for a time long enough to support liver functions in ALF patients till a tissue compatible transplant organ is available (or the own liver regenerates), and to reliably and consistently simulate the natural liver response to a drug challenge. It is generally accepted that the ideal bioreactor design to reliably simulate liver functions in vitro has to fulfill a few minimal requirements. Briefly, it should: provide cells with physiological supply and gradients of nutrients and gases; provide cell scaffolding and suitable cell-scaffold and cell–cell interactions such that cells may organize in space, polarize and re-differentiate as in the natural liver; permit convective flows to uniformly exchange metabolites and nutrients throughout the tissue mass; permit the physiological exchange of paracrine and autocrine biochemical cues; induce liver-specific enzymatic activities in parenchymal cells; maintain cells as viable and functional long-term; host a cell mass consistent with the intended application, e.g., a large cell mass for clinical applications or a minimal cell mass, yet representative of liver tissue behavior, for high-throughput preclinical drug screening. The complexity of liver metabolism, and the need for real-time correction of the metabolic imbalance means that in such 3D bioreactors, liver cells must exhibit near-physiological biotransformation of endogenous and exogenous substrates to bridge ALF patients to liver transplantation [8], or for the system to represent a feasible in vitro alternative to animal models in drug screening and to permit reliable scale-up of the in vitro drug clearance to that in vivo [9].

The metabolic competence of liver cells cultured in various 3D bioreactor designs, and the long-term retention of metabolic functions, is often assessed in terms of the disappearance rate of a drug or a parent compound, and the formation rate of their most relevant metabolites performed by the bioreactor as a whole. Such information is often used for scaling-up bioreactor design and performance for clinical or preclinical applications. A problem is that such rates are the compound effect of the intrinsic kinetics of the investigated liver cells metabolic reactions and the physical phenomena in which exogenous and endogenous species are involved in the bioreactor. Physical phenomena include species transport from the medium bulk to, and into, the cell aggregates, drug binding to proteins in medium, and drug adsorption on bioreactor constituents and tubing contacting medium [10,11,12]. Their effect on bioreactor performance largely depends on bioreactor configuration, geometry and materials, as well as on the operating conditions, and may disguise the intrinsic kinetics of drug biotransformation (i.e., unaffected by physical phenomena) by the liver cells cultured in the bioreactor. The cytochrome-P 450 (CYP) enzymes of the liver play a major role in the oxidative metabolism of foreign compounds. CYP450 enzyme activity is clinically assessed by challenging patients with a bolus of the drug lidocaine and by monitoring their liver’s capacity to eliminate it and to transform it to monoethylglicinexylidide (MEGX). Similarly, the CYP activity of liver cells cultured in vitro in various bioreactors is assessed with a lidocaine challenge. To the best of our knowledge, the effect of lidocaine transport and adsorption in the bioreactors is generally overlooked. This makes the kinetic information obtained depend on the specific bioreactor configuration and operation.

To overcome this limit, in this study, we report a retrospective analysis of the kinetics of lidocaine transformation to MEGX by porcine liver cells cultured around a 3D hollow fiber membrane network in four-compartment perfusion bioreactors with integral oxygenation. The bioreactors were operated under conditions minimizing metabolite transport resistance to/from the cells and ensuring a uniform distribution of matter in the bioreactor. Adsorption in the bioreactors was effectively accounted for with suitable kinetic modeling to extract the intrinsic kinetics of lidocaine biotransformation from the whole bioreactor performance.

## 2. Materials and Methods

### 2.1. Materials

**Figure 1:**
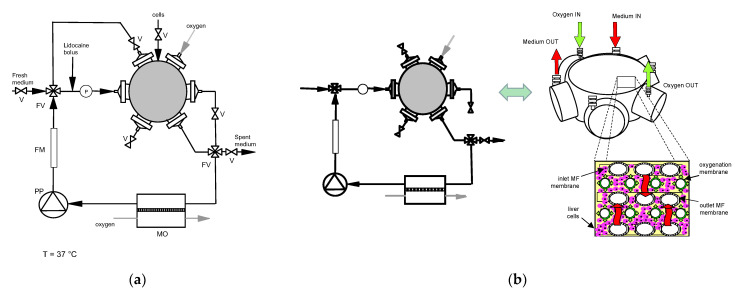
Three-dimensional bioreactor and experimental apparatus: (**a**) scheme of the experimental apparatus used for culture and kinetic experiments with the 3D bioreactors: FM—flowmeter; FV—four-way valve; MO—membrane oxygenator; PP—peristaltic pump; V—valve; (**b**) scheme of apparatus and 3D bioreactor operated in bleed-feed perfusion mode.

Three-dimensional bioreactors were used with a cell compartment of about 25 mL built according to the concept proposed by Gerlach et al. [13,14,15] (Figure 1) (StemCell Systems, Berlin, Germany). Briefly, the bioreactor core is a 3D membrane network consisting of a stack of alternating mats of orderly spaced, cross-woven microfiltration (MF) hollow fiber (HF) membranes made of polyethersulfone (inlet bundle) or polyamide (outlet bundle) for medical applications. Membranes in overlaid adjacent mats are aligned and angled at approximately 60 degrees with respect to one another, and are bundled with a separate inlet and outlet. Oxygen is supplied through a separate bundle of microporous polypropylene hollow fiber membranes for blood oxygenation. The 3D membrane network is encased in a polyurethane housing and each bundle is equipped with separate inlet/outlet headers. Hereinafter, such 3D bioreactors are referred to as bioreactors. Six-well tissue culture plates (Falcon, Becton Dickinson, and Company, Franklin Lakes, NJ, USA) pre-coated with collagen A (Biochrom, Berlin, Germany) were used as controls. To this aim, collagen A was diluted 1:1 with PBS supplemented with Ca^++^ or Mg^++^, and 0.5 mL of the diluted collagen solution was pipetted in each well. Collagen gelled after 1h incubation at 37 °C. Experiments were performed with Williams’ Medium E supplemented with 7% bovine fetal calf serum, amphotericine B (2.5 mg/L), penicillin (100,000 IU/L), streptomycin (100 mg/L), HEPES (0.01 mol/L), dexamethasone (80 µg/L), glucagon (2 µg/L) and pig insulin (16 IU/L), hereinafter referred to as medium. All chemicals were purchased from Biochrom (Berlin, Germany). Two-percent lidocaine (B. Braun Melsungen AG, Melsungen, Germany) was diluted to the desired challenge concentration with a physiological solution.

### 2.2. Lidocaine Adsorption Tests

Lidocaine adsorption on cell-free collagen-coated culture wells was characterized by incubation in lidocaine-containing medium for 6 h and by the timely collection of medium samples for analysis. After the tests, the wells were discarded. For the lidocaine adsorption tests with cell-free bioreactors, the bioreactors were primed with culture medium and were operated in the same apparatus and under the same conditions as in the kinetic tests with cell-seeded bioreactors, as described below. A lidocaine bolus was injected into the recycle loop, and medium samples were timely collected for 6 h for analysis. After the tests, the bioreactors were thoroughly rinsed with physiological solution and culture medium to wash out the adsorbed lidocaine and were used further for cell culture experiments. The medium samples were stored frozen until assayed for lidocaine and MEGX concentration.

### 2.3. Cell Isolation and Culture

Liver cells were isolated from anesthetized male, castrated piglets (German Landrace, 9–15 kg body weight) using the five-step collagenase perfusion technique described in previous papers [14] and were immediately put in culture (protocol approved by LAGeSo, Landesamt für Gesundheit und Soziales, Berlin, with number 0315/94, 20 September 1994). In the following, day 1 is the day when cells were first put in culture.

#### 2.3.1. Adhesion Culture

A total of 2.5 × 10^6^ freshly isolated porcine liver cells were seeded in each collagen-coated well. The wells were first rinsed with PBS supplemented with Ca^++^ or Mg^++^, then with medium containing 7% fetal calf serum. Seeded cells were allowed to adhere to collagen for 6 h. Then, unattached cells were removed by gentle rinsing with fresh culture medium, 2.5 mL fresh medium was added, and culture was started. Adherent cells were incubated at 37 °C, in a 95% air, 5% CO_2_, humidified gas mixture. Medium was exchanged daily. On various days after seeding, the adherent cells were challenged with lidocaine-containing medium to characterize the kinetics of lidocaine elimination and MEGX formation, as described below.

#### 2.3.2. Three-Dimensional Bioreactor Culture

The bioreactors were sterilized with ethylene oxide and degassed. Prior to cells seeding, they were extensively rinsed with medium to wash out any residual sterilant. For cell culture, each bioreactor was connected to a 125 mL recycle loop including a membrane oxygenator, as shown in Figure 1, and was integrated in a temperature-controlled chamber kept at 37 °C permitting control of the gas supply. Medical PVC tubing was used throughout. At the beginning of culture, about 6.108 freshly isolated porcine liver cells were injected into the bioreactor cell compartment, the bioreactor was rotated for 8 h to permit uniform cell distribution and attachment, and culture was started. For the first 16 h of culture, the bioreactor was operated in the bleed/feed “diffusion mode”. Fresh culture medium was continuously fed to (and spent medium removed from) the recycle loop at 12.5 mL/h while medium was continuously circulated around the bioreactor, and through the bore of both MF membrane bundles, at 45 mL/min. Under such conditions, metabolite transport to/from the cells occurs by diffusion and the cells are not subjected to shear stress, may recover from isolation, and may aggregate and form liver-like cell structures. After that, the three-way valves were switched in such a way to operate the bioreactor in the bleed/feed “perfusion mode” (Figure 1b) at a fresh medium feed flow rate of 9 mL/h and culture continued for 6 days. In the bleed/feed “perfusion mode”, the medium in the recirculation loop was fed to one end of the first MF membrane bundle while keeping the other end closed. Only one end of the second MF membrane bundle was kept open. This way, the medium entering the bore of the first MF membrane bundle was forced to permeate through the membrane walls, to flow outside and around the oxygenation membranes and through the cell mass, to be reabsorbed into the bore of the second MF membrane bundle and to leave the bioreactor through its open end. Under such conditions, metabolites transport to/from the cells is enhanced by convection and cells are subjected to fluid mechanical cues. Medium samples were collected timely from the recirculation loop through sterile filters to preserve bioreactor sterility. Medium pH and dissolved O_2_ and CO_2_ concentrations in the medium samples were measured with a type 178 blood gas analyzer (Corning, Halstead, Great Britain) and the operating conditions (e.g., gas composition and flow) were adjusted to maintain the circulating medium at pH = 7.2–7.45, pO_2_ = 180–200 mmHg, pCO_2_ = 20–40 mmHg. On day 2 and 6 of culture, the cells in the bioreactor were challenged with a lidocaine bolus to characterize the kinetics of lidocaine elimination and MEGX formation, as described below.

### 2.4. Kinetic Tests

The kinetics of lidocaine disappearance and MEGX formation were characterized starting on day 2 of culture. To this aim, the cells in adhesion culture were gently rinsed with fresh medium and were then incubated for 6 h with lidocaine-containing medium. The cell-seeded bioreactors were rinsed with two bioreactor volumes of fresh medium. Then, the fresh medium feed was stopped, the valves for medium influx and efflux to/from the recycle loop were closed, and the bioreactor was operated in closed-loop by recirculating the medium through the bioreactor at 45 mL/min (Figure 1b). The kinetic tests were started by injecting a lidocaine bolus into the circulating medium followed by culture for 6 h. In all cases, medium samples were collected timely and were stored frozen until assayed for lidocaine and MEGX concentration.

### 2.5. Analytical Methods

Immediately after isolation, an aliquot of cells was stored at −80 °C without the addition of cryoprotectant for CYP450 content determination. After thawing, cells were sonicated and CYP450 content was established photometrically from the CO-difference spectra of dithionate reduced samples according to Omura and Sato [16]. The total protein content of the cells was established according to Lowry et al. [17], and CYP450 content was expressed per unit total cell protein mass. Cell viability in adhesion cultures was assessed daily by visual examination of cell morphology under an inverted microscope (Zeiss, Unterkochen, Germany). The bioreactor construction hindered monitoring cell morphology by optical microscopy during culture. Hence, cell morphology in the bioreactors was assessed by histological analysis and light microscopy only at the end of the culture experiments. Histological sections were prepared by standard embedding techniques. Slices of the bioreactor content were stained with hematoxilin-eosin and according to Ladewig’s technique to identify connective tissue components of the extracellular matrix. Lidocaine and MEGX concentration in the culture medium was assessed by means of the TDxFLx fluorescence polarization immunoassay (Abbott, Wiesbaden, Germany). Their concentrations were corrected for the background noise for data analysis.

### 2.6. Data Analysis

**Figure 2:**
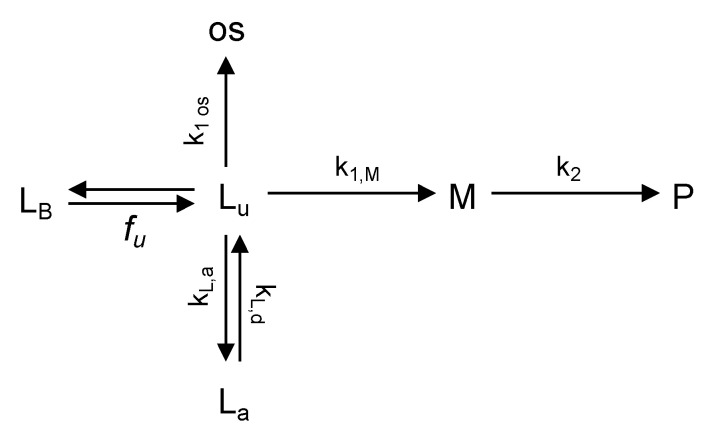
Scheme of metabolic and physical transformations, the kinetics of which was considered in the models proposed: La—adsorbed lidocaine; LB—protein-bound lidocaine; Lu—unbound lidocaine; M—MEGX; os—species other than MEGX formed from lidocaine; P—products formed from MEGX.

In modeling the metabolic and physical phenomena occurring in bioreactors and wells, it was assumed that lidocaine is transformed to MEGX and other species (e.g., 3-OH-lidocaine) and undergoes adsorption/desorption on/from bioreactor/well constituents. It was assumed that MEGX forms from lidocaine and undergoes further biotransformation (e.g., to glycinexylidine). The metabolic and physical phenomena considered are schematically shown in Figure 2. Mass balance equations for lidocaine and MEGX in the wells and in the bioreactors were obtained under the assumption that the metabolites distribute uniformly in medium (i.e., well mixed volume), as follows:

Lidocaine in medium:

(1) $$-\frac{dC_{L}}{dt}=-r_{L}=-\left(r_{M}+r_{L,os}+r_{L,a}\right)+r_{L,d}=$$

$$=-\left(r_{1}+r_{L,a}\right)+r_{L,d}$$

Lidocaine in the adsorbed phase:

$$\frac{dC_{L,a}}{dt}=r_{L,a}-r_{L,d}$$

MEGX in medium:

$$\frac{dC_{M}}{dt}=r_{M}-r_{2}$$

Subject to the following initial conditions:

I.C.  *t* = 0    *C_L_ = C_L,*0*_ C_M_* = 0 *C_L,a_* = 0.

It was also assumed that the dissolved oxygen concentration is constant during the kinetic tests and that metabolites other than lidocaine and MEGX have negligible effects on the kinetics of the investigated reactions. Kinetic models relating the rate of lidocaine metabolic disappearance and physical adsorption and of MEGX metabolic transformations to their concentrations were sought that would yield model-predicted lidocaine and MEGX concentrations in medium in agreement with those measured during the kinetic tests. If deemed useful, lidocaine in the adsorbed phase could be predicted by the model for the best-fit parameter values. Experimental data previously reported [18] were included in the analysis. Lidocaine binding to serum proteins was accounted for with an unbound lidocaine fraction fu = 0.65 [19]. Power law (i.e., *r_i_ = k_i_ C_i_^α^*) and Michaelian (i.e., *r_i_ = V_max,i_ C_k_/(K_M,i_ + C_k_*)) kinetic models were considered. The best-fit parameter values for each investigated model were obtained with a custom MATLAB software based on a modified Levenberg–Marquardt technique coupled to an ordinary differential equation solver to integrate the set of mass balance equations for lidocaine and MEGX in each culture system. The differential and the integral methods were used for seeking initial parameter guesses [20]. The best-fit models were selected as those minimizing the sum of squared residuals between experimental (*C_i,exp_*) and model-predicted (*C_i,mod_*) metabolite concentrations over the kinetic test time corrected for the number of model parameters, p, according to an extension to non-linear models of Akaike’s Information Criterion (AIC) [21], as follows:

(2) $$AIC=2\left(p+1\right)+n\;Ln\left(\frac{1}{n}\sum_{k=1}^{m}\sum_{j=1}^{n}{\left(C_{k,exp,j}-C_{k,mod,j}\right)}^{2}\right)\;=2\left(p+1\right)+n\;Ln\left(SSR/n\right)$$

where *n* is the number of data points, *m* the number of experiments, and $SSR=\overset{m}{\underset{k=1}{\sum }}\overset{n}{\underset{j=1}{\sum }}{\left(C_{k,exp,j}-C_{k,mod,j}\right)}^{2}$ is the sum of squared residuals.

The bioreactor capacity to culture cells in a physiological microenvironment and to expose them to physiological lidocaine and metabolite concentration profiles following the lidocaine challenge was characterized in terms of the MEGX index, that is, the MEGX-to-lidocaine concentration ratio at any time during the kinetic test.

Data are generally reported as mean +/− standard deviation. The statistical significance of concentration differences in the medium recirculating in the bioreactors during the kinetic tests was assessed with the Student’s *t*-test after checking that the data distribution is normal.

## 3. Results

### 3.1. Lidocaine Adsorption in Cell-Free Bioreactors

**Figure 3:**
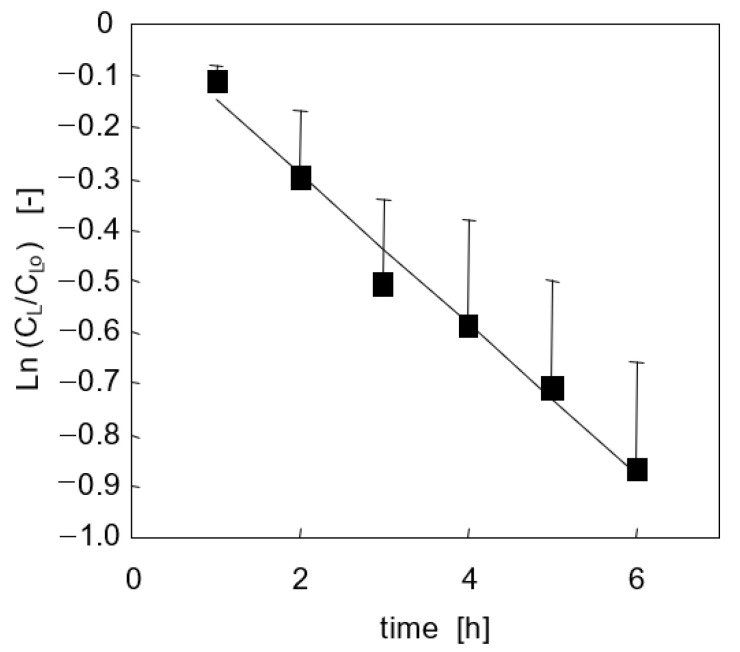
Lidocaine disappearance from medium in adsorption tests with cell-free bioreactors (*n* = 7). Line is model prediction.

Lidocaine concentration in the medium of cell-free collagen-coated wells did not change significantly over 6h incubation at 37 °C, suggesting that lidocaine adsorption is negligible. Following the bolus injection in the medium of cell-free bioreactors, lidocaine concentration decreased exponentially with time, as shown in Figure 3. Data analysis suggests that lidocaine disappears from the medium at a rate proportional to its unbound concentration (i.e., *–r_L,a_ = k_L,a_ f_u_ C_L_*) with an adsorption constant *k_L,a_* = 0.26 h^−1^. MEGX was not detected in the medium of either culture system.

### 3.2. Lidocaine Disappearance in Cell-Seeded Bioreactors

Cell viability ranged from 95 to 99% as determined by trypan blue exclusion. The amount of CYP found in the porcine liver cells was 0.28 nmol_CYP_/mg_protein_, comparable to other pig breeds [22,23].

#### 3.2.1. Adhesion Culture

**Figure 4:**
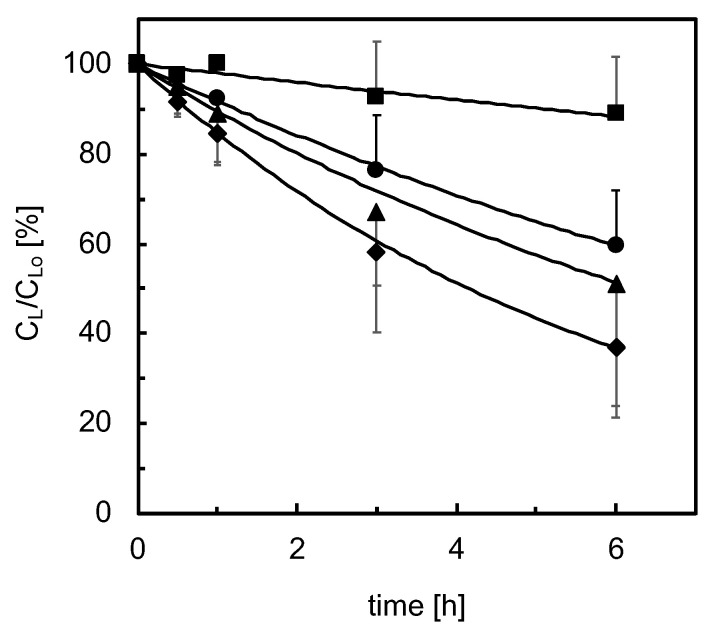
Lidocaine elimination during kinetic tests with adhesion cultures (*n* = 3) at various days of culture: (♦) day 2; (▲) day 3; (●) day 5; (■) day 14. Lines are model predictions.

**Figure 5:**
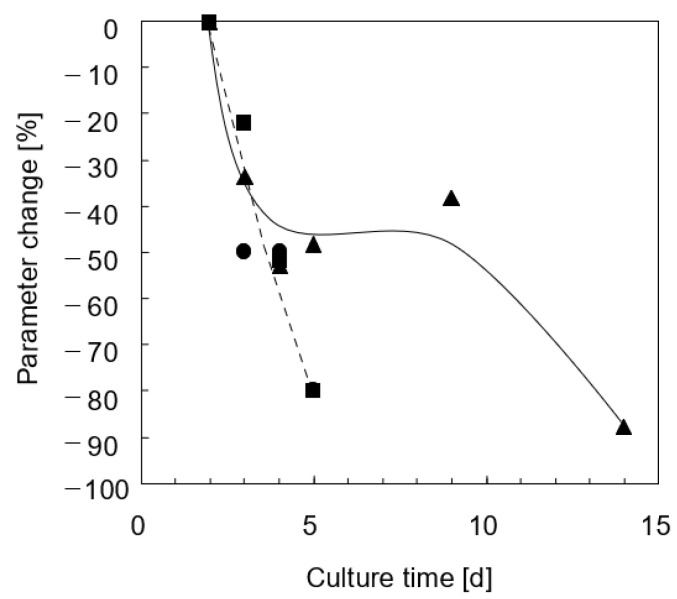
Time decay during culture of the transformation kinetic constants with adherent cells: (▲) *k_*1*,L,A_* for lidocaine elimination; (●) *k_*1*,M,A_* for MEGX formation; (■) *k_2,A_* for MEGX further transformation.

**Figure 6:**
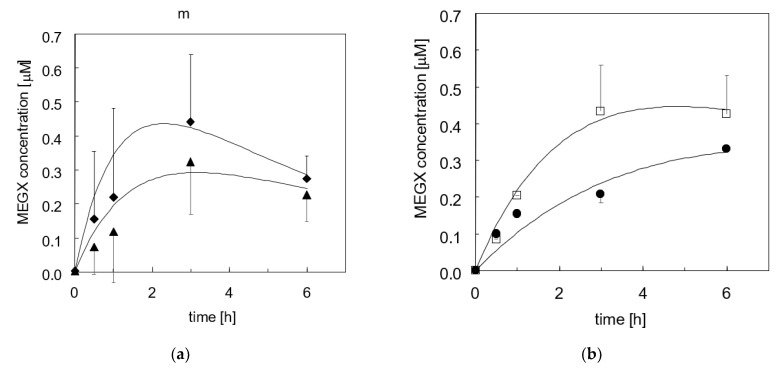
MEGX concentration profile in time during the kinetic tests (*n* = 3) with adherent cells at various days of culture: (**a**)—(♦) day 2; (▲) day 3; (**b**)—(□) day 4; (●) day 5. Lines are model predictions.

Cells adhered within 4–6 h from seeding, spread and formed a confluent monolayer. During the first week of culture, analysis by light microscopy did not evidence any damage to the cell wall. As is often the case for 2D culture, at longer times, a reduction of the intercellular connections was observed, eventually followed by cell detachment from the support. In the kinetic tests, lidocaine concentration in medium significantly decreased in exponential fashion in time after the lidocaine bolus at a rate that decreased with increasing culture times, as shown in Figure 4. Data analysis suggests that lidocaine disappears at a rate proportional to its unbound concentration (i.e., *−r_L,A_ = k_*1*,A_ f_u_ C_L_*). On day 2 of culture, the kinetic constant of lidocaine disappearance is *k_*1*,A_* = 0.26 h^−1^, then it decreases with time, levels off from day 4 to day 9 at a value about 50% of that on day 2, and sharply decreases at longer culture times, as shown in Figure 5. Lidocaine disappearance exceeded the amount of MEGX formed, suggesting that lidocaine is metabolically transformed also to other species at a rate proportional to the unbound lidocaine concentration (i.e., *−r_L,A_ = k_*1*,A_ f_u_ C_L_ = (k_*1*,M,A_ + k_*1*,os,A_) f_u_ C_L_*). Data analysis suggests that the MEGX produced from lidocaine is further transformed to other metabolic products. Figure 6a shows that, during the kinetic tests at culture day 2 and 3, MEGX concentration initially increases with time, peaks up after about 2–3 h and then decreases with a bell-shaped curve as a result of other serial transformations.

Figure 6b shows that in tests performed at day 4 or later, the MEGX concentration profile in time gradually lost its bell shape. Data analysis suggests that MEGX forms from lidocaine at a rate proportional to the unbound lidocaine concentration and it is transformed to other metabolites at a rate proportional to its concentration, yielding the following equation for the net rate of MEGX formation: *r_M,A_ = k_M,A_ f_u_ C_L_ − k_2,A_ C_M_*. At culture day 2, the kinetic constants were *k_M,A_* = 4.3 × 10^−*2*^ h^−1^ and *k_*2*,A_* = 0.89 h^−1^, respectively. Both constants steeply decreased with culture time at about the same rate, as shown in Figure 5. The cell transformation capacity in one well sharply decreased on day 5 indicating a serious loss of viability, and that culture was terminated. From culture day 9 on, cells slowly eliminated lidocaine but MEGX concentration could not be reliably detected.

#### 3.2.2. Three-Dimensional Bioreactor Culture

**Figure 7:**
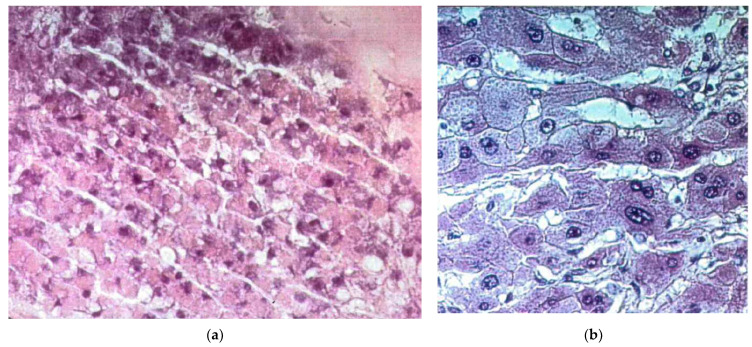
Histological sections of liver tissue from a 3D bioreactor and a patient with liver cirrhosis stained with hematoxylin and eosin: (**a**) tissue from a 3D bioreactor after 7 days of culture (magnification 320×); (**b**) tissue from the liver of a cirrhotic patient (magnification 400×). Details on tissue preparation can be found in the Section 2 Materials and Methods Section.

Histological analysis of cell organization in the 3D bioreactors was performed at the end of culture. The histological sections (Figure 7) showed that the liver cells had mainly formed thick aggregates stretching through and partially filling the gaps between neighboring HF membranes. Aggregates a few cells thick coating the HF membrane outer surface were also observed. Cells organization and the formation of canaliculi in the aggregates was similar to that in cirrhotic livers, where injury to parenchymal and non-parenchymal cells induces cell proliferation and re-organization.

**Figure 8:**
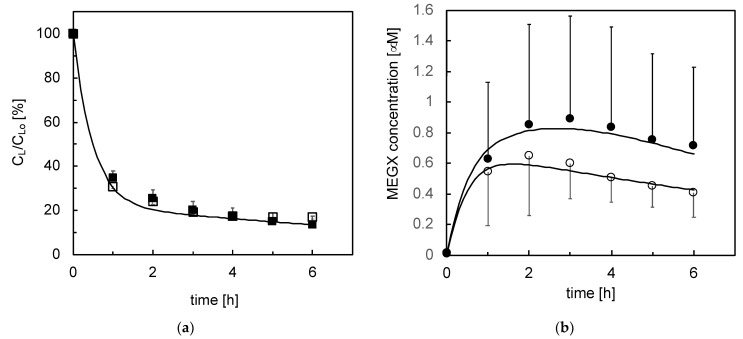
Metabolite concentration profiles in time during the kinetic tests with cell-seeded bioreactors (*n* = 8) at different days of culture: lidocaine ((**a**), □) and MEGX ((**b**), ○); open symbols—day 2; closed symbols—day 6. Lines are model predictions.

**Figure 9:**
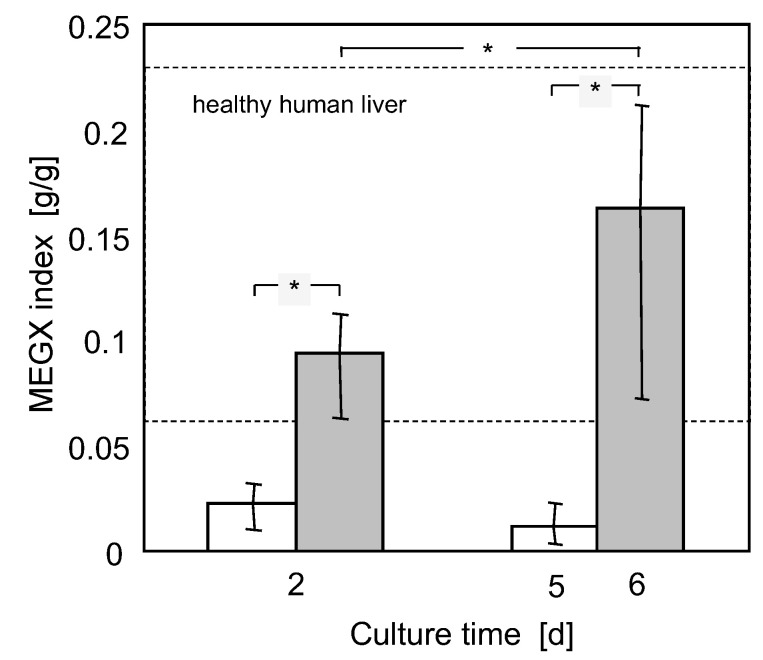
Average MEGX index at various days of culture for cells in adhesion (white bars) or bioreactor (grey bars) culture. Error bars indicate the minimal and maximal values. The shadowed region shows the MEGX index range for healthy human liver. *—statistically significant differences (*p* = 0.05).

In the kinetic tests, lidocaine concentration in medium decreased exponentially with time following the bolus injection and almost leveled off after about 4–6 h, as shown in Figure 8. Data analysis suggests that lidocaine is metabolically eliminated at a rate proportional to its unbound concentration in medium (i.e., −*r_L,B_ = (k_*1*,M,B_ + k_*1*,os,B_*)* f_u_ C_L_*), and undergoes reversible Langmuir-type adsorption in the bioreactor (i.e., *−r_a,B_ = k_L,a_ f_u_ C_L_ − k_L,d_ C_L,a_*). The kinetic constant of lidocaine disappearance by cell metabolism and physical adsorption (i.e., *k*_1,*B*_ *= k*_1*,M,B*_ *+ k*_1,*os,B*_ *+ k_L,a_*) is about constant at *k*_1,*B*_ = 2.3 h^−1^ on day 2 and 6. The lidocaine adsorption constant slightly decreases from *k_L,a_* = 1.8 h^−1^ to *k_L,a_* = 1.6 h^−1^, and the desorption constant increases from *k_L,d_* = 0.52 h^−1^dm^−1^ to *k_L,d_* = 0.84 h^−1^dm^−1^ at day 2 and 6, respectively. In one experiment, the pressure upstream from the bioreactor became very high. To avoid mechanical damages to cells, but not to stop culture, pressure was lowered by diverting part of the medium entering the bioreactor through the cell seeding. Figure 8a,b shows that, although rather scattered, MEGX concentrations initially increased with time, peaked up and then decreased with a bell-shaped curve. The time after the lidocaine challenge at which MEGX concentration peaks up was shifted from about 2 to 3 h from day 2 to 6, respectively. Data analysis suggests that lidocaine is metabolically transformed to MEGX at a rate linearly dependent on the unbound lidocaine concentration and that MEGX is further transformed to other metabolites at a rate proportional to its concentration yielding the following equation for the net rate of MEGX formation: *r_M_ = k*_1,*M,B*_ *f_u_ C_L_ − k*_2,*B*_ *C_M_*. The kinetic constant of MEGX formation from lidocaine is about constant at *k*_1,*M,B*_ = 8.8 × 10^−2^ h^−1^ at both day 2 and 6. The rate at which lidocaine is transformed to species other than MEGX increased during culture. The kinetic constant of such transformation at day 6 is about 1.6 times higher than the *k*_1*,os,B*_ = 0.44 h^−1^ at day 2. The kinetic constant of MEGX transformation to other metabolites at day 6 is about 56% of the *k*_2,*B*_ = 0.5 h^−1^ value at day 2. Figure 3, Figure 4, Figure 5 and Figure 6 and 8 show that the model-predicted lidocaine and MEGX concentrations agree rather well with the experimental results, suggesting the goodness of the analysis proposed. Figure 9 shows that, in the course of the kinetic experiments, during the whole lidocaine challenge, the MEGX index varied but consistently remained in the physiological range for healthy liver for cells in bioreactor culture, and below such range in adhesion culture.

## 4. Discussion and Conclusions

In Western countries, a large number of candidates for liver transplantation develop ALF as a consequence of drug intoxication. Lack of detoxification and accumulation of toxic substances is associated to changes in the mental status of patients with hepatic encephalopathy [8]. Hence, an important BAL feature is its capacity to detoxify drugs from the patient’s blood. Similarly, in the development of new therapeutic drugs, liver metabolic reactions play an important role in drug clearance, which has pharmaceutical and toxicological implications. Among the enzymes that metabolize foreign compounds, the cytochrome-P (CYP) 450 enzymes constitute a superfamily that plays a major role in the oxidative metabolism of toxic chemicals, endogenous biochemicals, hormones and drugs [24]. Obtaining equations correlating to the elimination rate of a drug to its concentration and the liver cell mass would permit to rationally decide the liver cell mass that should be loaded in a BAL to stabilize an ALF patient and to predict the in vivo clearance of a drug from in vitro experiments.

In this study, porcine liver cells were cultured around a perfused 3D hollow fiber membrane network with integral oxygenation in a scaled-down version of a four-compartment bioreactor design proposed for the treatment of ALF patients [8]. Because of the anatomical and physiological similarity to humans, porcine livers and tissues have been used in research on xenotransplantation, ex vivo liver perfusion, and bioartificial livers to develop strategies to treat ALF patients [25,26,27]. Today, in many countries, the fear of disease transmission from pigs to humans prevents the use of porcine tissue for therapeutic treatments, yet porcine tissue is a good model of human tissue. Pigs are also considered feasible animal models in pharmacological and toxicological studies [28,29,30]. Enzymatic activities analogous to human CYP1A, CYP2A, CYP2B, CYP2C, CYP2D, CYP2E and CYP3A have been found or biochemically induced in various pig breeds [4,5,22,23,28,29,30,31,32,33,34]. However, limited information is available on CYP450 activity in porcine liver cells compared to other species [35]. Studies on isolated pig liver cells cultured in adhesion on flat substrata or in 3D scaffolds, in static culture dishes or in perfusion bioreactors, have shown that the CYP450 enzymes expressed in cells and their capacity to eliminate drugs long-term depend on the culture type and on bioreactor design and operation [26,27,28,29,30,36,37,38,39]. Such capacity is generally characterized in terms of the rate at which the bioreactor used for cell culture eliminates a drug from a given medium volume under given operating conditions.

The bioreactors used for this study have advantageous features. The membrane scaffolding, the decentralized and uniformly distributed oxygen supply enabled by the interwoven oxygenation membranes, and controlled cell perfusion with medium yields near-to-physiological solutes gradients and permits the re-organization of parenchymal and non-parenchymal primary adult and fetal liver cells in 3D aggregates similar to natural liver tissue and the expression of markers of adult hepatocytes, endothelial, Kuppfer, biliary and even liver stem cells, and promotes hepatic and endothelial differentiation of immature cells [40,41,42,43]. Moreover, liver cells cultured in such bioreactors remain viable, synthesize proteins, and metabolize drugs for up to 30 days [44,45,46].

In this study, the drug clearance capacity of porcine liver cells was characterized with respect to lidocaine transformation to MEGX. Lidocaine is a widely used local anesthetic and anti-arrhythmic amide-type drug. In the human liver, lidocaine is mainly metabolized by CYP450 enzymes to MEGX [47,48] at rates significantly reduced in individuals with liver diseases [49,50]. For this reason, lidocaine transformation to MEGX following intravenous injection of a lidocaine bolus is clinically used as a marker of CYP450 activity in the liver and as a predictor of the liver healing potential [51,52,53]. The mechanism of lidocaine metabolism in pigs is not as well understood as in humans. Sielaff et al. [30] have reported that liver cells from Dorac male pigs cultured in cylindrical gels eliminate lidocaine and, like human cells, form MEGX, 3-OH-lidocaine, 4-hydroxy-2,6-dimethylaniline and its glucuronide. MEGX is further transformed to 3-OH-MEGX and glycinexylidine but not to xylidine. So far, the absence of quantitative standards has precluded quantitative conclusions. Researchers often report only on MEGX formation from lidocaine as a means of characterizing the long-term CYP450 activity of pig liver cells in different culture models. Reports on structure and activity of CYP450 enzymes in pig liver tissue generally lack consistency and evidence significant differences for different strains or breeds and among individuals [4,23,54]. Only a few rate equations have been reported for isolated porcine liver cells, which generally correlate their oxygen consumption rate to the dissolved oxygen partial pressure in medium [55].

Uncertain information and general acceptance of the MEGX test in the clinics made us decide to characterize only the kinetics of lidocaine transformation to MEGX. In fact, to the best of our knowledge, no rate equation for lidocaine elimination to MEGX by primary porcine liver cells has been reported yet. Tests were performed in the lidocaine concentration range (about 5–20 μM) to which patients are exposed when tested for their liver conditions or in clinical applications [56,57,58] to account for the different dependence on lidocaine concentration of the metabolic transformation rates by different enzymes [59,60].

It is worth noting that neglecting the resistance to metabolites transport and assuming that medium is well mixed in the bioreactors does not limit the goodness of model predictions. In fact, we showed previously that an inert tracer distributes uniformly in these bioreactors in a time shorter than the total bioreactor volume (i.e., including tubing)-to-recirculation flow rate ratio [61]. In this study, such ratio is negligible with respect to the characteristic times of the investigated cellular reactions. This supports the assumption that medium in the bioreactors is well mixed and that the resistance to metabolites transport may be neglected.

### 4.1. Lidocaine Adsorption

Lidocaine adsorption was negligible in the collagen-coated culture wells, and its effects could be neglected in the analysis of adhesion culture data. Consistent with previous reports [12], significant amounts of lidocaine adsorbed on the constituents of bioreactors and the circulation loop. Lidocaine adsorbed to a greater extent in the presence of cells, accounting for an average of 72% of the overall specific rate (i.e., the kinetic constant) of lidocaine disappearance. This is consistent with that reported by Obach [62] and Shibata et al. [63], who have shown that lipophilic basic drugs, such as lidocaine, substantially bind to hepatic microsomes and liver cells. Liver cell aggregation in overlaid layers and medium perfusion in the bioreactors both contribute to making it more likely for unbound and protein-bound lidocaine to get physically adsorbed on, or trapped in, the MF membrane pores and the dense cell aggregates, which could act as sieves. The pressure increase occasionally observed upstream from the bioreactors confirms that during culture, proteins adsorb onto the membranes, which may decrease their hydraulic permeability. Lidocaine physically sequestered by any means is unavailable for direct interaction with metabolizing enzymes and must dissociate from the non-specific adsorption bond to be metabolized. When lidocaine concentration in medium decreases, the adsorbed lidocaine may be desorbed and transported back into the medium, balancing out the rate at which lidocaine is metabolized. This may have caused the almost constant lidocaine concentrations from 4h shown in Figure 7 and Figure 8, which is correctly predicted by the kinetic model. The decrease of *k_L,a_* and the increase of *k_L,d_* on day 6 is consistent with the reduced lidocaine adsorption observed in bioreactors after repeated exposure of bioreactor materials to medium supplemented with serum [12]. This is possibly caused by the non-specific adsorption of serum proteins on the materials that progressively reduces the available adsorption sites for lidocaine. The extent of the adsorption/desorption phenomena evidences the importance of accounting for their occurrence when culture experiments are performed to characterize the liver cell capacity to metabolize lidocaine. It is only in this way that information on the intrinsic cell metabolism may be extracted from the measured bioreactor capacity to eliminate lidocaine or other drugs. These also suggest that when the bioreactors are used to assist ALF patients, adsorption may significantly boost the bioreactor capacity to remove toxins from the patient’s plasma beyond that permitted by the liver cell metabolism.

### 4.2. Lidocaine Metabolic Elimination

Various membrane bioreactor configurations have been proposed for liver cell culture as the core of a bioartificial liver [64]. To the best of our knowledge, this is the first quantitative characterization of lidocaine elimination kinetics in one of such bioreactors. This is also the first study in which the distribution of lidocaine in the bioreactor was optimized for the purpose, and the kinetics of lidocaine adsorption was characterized. For this reason, the results obtained are only compared to that reported on 2D cultures of liver constituents.

In the 3D bioreactors, the liver cells eliminated lidocaine at a rate proportional to the unbound lidocaine concentration in both bioreactors and adhesion cultures. Such dependence hints at a similarity with human tissue. In fact, the rate of lidocaine elimination by human liver microsomes has been reported to depend on lidocaine concentration according to a Michaelis–Menten-type equation with K_M,L_ values ranging from 0.37 to 3 mM; the lower the value, the worse the liver conditions [50]. The lidocaine concentration challenge used for this study is far lower than such *K_M,L_* values. This would make the Michaelian rate equation collapse in the first-order rate equation observed in this study, as it would occur with human liver tissue. Data analysis yielded sound values of the cell-specific kinetic constants for the metabolic lidocaine elimination (i.e., *k’*_1_ *= k’*_1,*M*_ *+ k’*_1,*os*_) in agreement with that reported for perfused isolated porcine livers [65]. In fact, assuming a 1.03 × 10^8^ cells/g_liver_ cell concentration in the liver [66] and a 1 g/mL liver density [67], lidocaine would be eliminated by pig livers with a cell-specific kinetic constant of about *k’*_1_ = 2.3 × 10^−7^ mL/(n_cell_ h) [65]. Such value is in good agreement with those estimated in this study for cells cultured up to day 6 in bioreactors (i.e., *k’*_1,*B*_ = 1.3–1.9 × 10^−7^ mL/(n_cell_ h)) and adhesion cultures (i.e., *k’_1,A_* = 1.3–2.6 × 10^−7^ mL/(n_cell_ h)). Interestingly, at day 2, the cell-specific kinetic constant for lidocaine metabolic elimination in bioreactors *k’*_1,*B*_ was about a half of that in adhesion culture *k’*_1,*A*_, but at day 6, *k’*_1,*B*_ increased about 1.5 times, whereas *k’*_1,*A*_ decreased to a half of that at day 2. The type of culture did not have the same effect on all enzymatic activities involved in the metabolism of lidocaine and its metabolites. In fact, the kinetic constants of MEGX formation from lidocaine *k*_1,*M*_ and further biotransformation, *k_2_*, reactions at day 2 were both higher for cells in adhesion culture than in bioreactors. Although initially lower, the kinetic constants of all metabolic transformations of cells cultured in the bioreactors remained about constant up to day 6, suggesting that the bioreactor culture helps maintain stable liver cell enzymatic activities for the investigated time. The amount of CYP in the cells consistent with that of other mammals and the values of *k’*_1,*A*_ and *k’*_1*,B*_ at day 2 consistent with that of perfused pig livers suggest that the enzymes involved in lidocaine metabolism were initially present in the cells in proper amounts. Oxidative stress during and following cell isolation or hypoxic conditions in the early culture times possibly damaged some of the CYP activities in enzyme-specific fashion [68,69]. For the cells in adhesion culture, Figure 4 shows that the kinetic constants of all considered reactions steeply decreased in the first days of culture with a 50 to 80% loss on day 5 of the enzyme activities involved in lidocaine and MEGX further transformation, respectively. Enzyme activity losses are possibly to be blamed by the insufficient supply to cells of oxygen from the gas phase above the medium [68]. The dissolved oxygen concentration at the cell surface may be estimated from the pseudo steady-state mass balance of dissolved oxygen in the stagnant medium above cells metabolizing oxygen with Michaelian kinetics as follows:

(3) $$C_{O_{2}}=\frac{1}{2}\;[{\left(K_{M,O_{2}}-C_{O_{2},eq}+Da\;K_{M,O_{2}}+4\;K_{M,O_{2}}C_{O_{2},eq}\right)}^{\frac{1}{2}}\;-(K_{M,O_{2}}-C_{O_{2},eq}+Da\;K_{M,O_{2}})]$$

with $Da=\frac{k_{O_{2}}^{’}\;C_{cell}\;h}{K_{M,O_{2}}\;D_{O_{2}}}$.

For porcine liver cells, Balis et al. [55] estimated that *k’*_*O*_2__ ranges from 9.1 × 10^−7^ nmol/(cell min) to 3.1 × 10^−7^ nmol/(cell min), and *K_M,O_*2*__* from 1.9 to 4 mmHg in the early culture and the post-attachment phase, respectively. For *D*_*O*_2__ = 2 × 10^−5^ cm^2^/s, *C_cell_*= 2.6 × 10^−5^ cells/cm^2^, *C*_*O*_2_*,eq*_ = 19 nmol/mL, and *h* = 0.26 cm (see Section 2 Materials and Methods), Equation (3) yields a dissolved oxygen concentration at the cell surface of a few mmHg’s in the early culture times, and of 25 mmHg thereafter, well below the 35 mmHg value for perivenous blood. Persistent effects of oxidative stresses caused by cell isolation cannot be ruled out. After the first days of culture, the kinetic constants decreased in time in a different fashion, suggesting that the enzymes involved in the metabolic reactions of lidocaine and its metabolites exhibit a different capacity to withstand hypoxia. The kinetic models predicted fairly well the change during culture of the time at which MEGX concentration peaks up (i.e., *t_max_*). The model predicts that the kinetic constant of the MEGX further biotransformation (i.e., *k*_2,*A*_, *k*_2,*B*_) decreases in time faster than that of MEGX formation from lidocaine (i.e., *k*_1,*A*_*, k*_1,*B*_). This causes the shift towards longer *t_max_* and eventually causes the MEGX concentration profile to lose its typical bell shape with downward concavity as cells die off in adhesion culture, as shown in Figure 5 and Figure 8 [18]. Maintenance of a good liver cell state and competence in the bioreactor is possibly permitted by the favorable cell microenvironment produced by the controlled intermembrane spaces enabled by the orderly membrane arrangement and by the bleed/feed perfusion mode.

Considering the fact that cells seldom formed large aggregates, the distributed oxygen supply through the cell mass and the enhancement of oxygen transport caused by medium perfusion likely contributed to establishing oxygen concentration gradients across the cell aggregates that better replicate those in the liver acinus while providing sufficient oxygen to the cells. An important requirement for cell culture systems aiming to mimic the drug clearance capacity of the natural liver is that the drug and its metabolites concentrations are distributed in a similar pattern to the natural liver to ensure that cells are exposed to physiological metabolic challenges. The extent to which adsorption influenced lidocaine elimination prevents from using the MEGX index as a marker of the liver cell state. Nonetheless, the MEGX index may provide information on the lidocaine concentrations to which cells are exposed during the kinetic tests relative to MEGX concentrations. Figure 9 shows that the MEGX index for cells cultured in the bioreactors consistently was within the range typical of healthy human livers, whereas it was lower than that in adhesion culture. Among the 3D membrane bioreactors proposed for BALs, the MEGX index could only be estimated for liver cells cultured in the AMC bioreactor [38]. In such bioreactor, liver cells are entrapped in a wound 3D polyester nonwoven fabric in which hollow fiber membranes for blood oxygenation are axially inserted to enhance oxygen transport to cells. When tested for lidocaine elimination, the liver cells in the AMC bioreactor yielded values of the MEGX index ranging approximately from 0.05 to 0.09, at the lower limit of healthy human livers. All this provides additional evidence that cells in the 3D bioreactors used for this study are cultured in a more physiological microenvironment than that offered by adhesion cultures or such a promising bioreactor as the AMC bioreactor. As for many 3D bioreactors proposed for liver cell culture, the main limitation to using these 3D bioreactors for pharmacological studies is possibly that their construction, albeit convenient for the cells, hinders the analysis of cell morphology and organization during culture by optical microscopy. Preparation of the histological sections also requires qualified personnel trained for the purpose.

In conclusion, this study characterized the kinetics of the biotransformation reactions of lidocaine and some of its metabolites by porcine liver cells cultured in 3D membrane network bioreactors. The reported analysis suggests that the bioreactors are feasible for assistance to ALF patients and for in vitro preclinical screening. In fact, as a result of cell organization and bioreactor operation, lidocaine elimination metabolic activities are stable for about a week, and cells are exposed in time to near-physiological lidocaine and MEGX concentration profiles when subjected to a lidocaine challenge. The analysis also suggests that physical adsorption and transport phenomena in a given bioreactor should be properly coupled to the intrinsic kinetics of cellular metabolic reactions to design therapeutically effective BALs and to effectively predict the in vivo liver clearance of a drug from in vitro experiments with liver cell models.

## Data Availability

Data is contained within the article.

## Nomenclature

*C*	solute concentration, M
*C_cell_*	cell density, cell/cm^2^
*C_L,a_*	lidocaine concentration in the adsorbed phase, moles/cm^2^
*C_O_*2*_,eq_*	dissolved oxygen concentration in medium equilibrating the above gas, M
$Da=\frac{k_{O_{2}}^{’}\;C_{cell}\;h}{K_{M,O_{2}}\;D_{O_{2}}}$	Damkoehler number,
*D_O_*2*__*	oxygen diffusion coefficient in medium, cm^2^/s
*f_u_*	unbound lidocaine fraction,
*h*	medium thickness above cell surface in adhesion culture, cm
*k_i_*	kinetic constant for the i-th transformation, s^−1^
*k_i_’ = k_i_/C_cell_*	cell-specific kinetic constant for the i-th transformation, cm^3^/(s n_cell_)
*K_M_*	Michaelis constant, M
m	number of experiments,
n	number of data points,
p	number of model parameters,
-r	metabolite disappearance rate, M/s
*Subscripts/Superscripts*	
a	adsorption
d	desorption
exp	experimental
i	i-th metabolite
j	j-th data point
k	k-th experiment
mod	model-predicted
os	species other than MEGX produced by lidocaine metabolic transformation
A	adhesion culture
B	bioreactor culture
L	lidocaine
M	MEGX
O_2_	dissolved oxygen
0	initial value
1	lidocaine metabolic transformation
2	MEGX further metabolic transformation
α	reaction rate order
